# 13124 COVID-19: The Urgency of Engaging during Crisis

**DOI:** 10.1017/cts.2021.594

**Published:** 2021-03-30

**Authors:** Krista Bohn, Sharon Croisant, Lance Hallberg, John Prochaska, Chantele Singleton

**Affiliations:** University of Texas Medical Branch

## Abstract

ABSTRACT IMPACT: Throughout the COVID-19 pandemic, the UTMB Institute for Translational Sciences has sought to answer our communities’ needs for research, for knowledge of research, and involvement in research, while recognizing that meaningful engagement involves understanding all emergent needs and responding to maximize the health and well-being of those we serve. OBJECTIVES/GOALS: ITS community programs responsive to COVID-19 include:

ο Ongoing communication with community and business stakeholders

ο Social media and public health campaigns promoting safe practices, research updates, and testing information

ο Community initiatives to increase testing among vulnerable populations METHODS/STUDY POPULATION: Like sister hubs across the US, the UTMB ITS has brought available resources to bear on addressing COVID-19 through research, medical response, and public health outreach. Community engagement activities have included facilitating communication, particularly by rapidly translating information for multiple audiences and wherever possible and appropriate, providing opportunities for the patient’s voice to inform and guide development of research. We realized the community’s need for trustworthy and reliable information about COVID-19 early in the pandemic. Key partnerships with community members and organizations were critical in enabling us all to be most responsive in meeting these needs. RESULTS/ANTICIPATED RESULTS: ITS community outreach included developing infographics, media notices, and educational materials related to prevention and testing as well as appropriate use of PPE. These efforts resulted in an article in a regional newspaper, which was disseminated widely through social media networks. ITS faculty also engaged doctoral and MPH trainees to support the Health District’s contact tracing effort. We held several events on mental health impacts as well as discussions related to health disparities. Both activities shaped plans for community-based interventions and research. The ITS also hosted a virtual workshop to facilitate discussion around key research questions related to the pandemic. DISCUSSION/SIGNIFICANCE OF FINDINGS: Throughout the pandemic, the ITS has maintained contact with stakeholders. Our roles have been to communicate, disseminate, translate, provide resources, and build bridges. We also listen, share, and provide opportunities for patients and communities to engage in all phases of the research spectrum.

